# Impact of COVID-19 Pandemic on Tourist Travel Risk Perception and Travel Behaviour: A Case Study of Poland

**DOI:** 10.3390/ijerph20085545

**Published:** 2023-04-17

**Authors:** Anna Jęczmyk, Jarosław Uglis, Jan Zawadka, Joanna Pietrzak-Zawadka, Monika Małgorzata Wojcieszak-Zbierska, Magdalena Kozera-Kowalska

**Affiliations:** 1 Department of Law and Enterprise Management in Agribusiness, Faculty of Economics, Poznań University of Life Sciences, 60-637 Poznań, Poland; 2 Department of Tourism, Social Communication and Counselling, Institute of Economics and Finance, Warsaw University of Life Sciences—SGGW, 02-787 Warsaw, Poland; 3 Institute of Forest Sciences, Faculty of Civil Engineering and Environmental Sciences, Bialystok University of Technology, 15-351 Bialystok, Poland; 4 Department of Economics and Economic Policy in Agribusiness, Faculty of Economics, Poznań University of Life Sciences, 60-637 Poznań, Poland

**Keywords:** tourism, travel risk, COVID-19, consumer behaviour, travel market, economic decision

## Abstract

This article aims to identify the impact of the COVID-19 pandemic on the perception of the risk of travel and travel behaviour by Poles. The study was conducted using the survey method and the CAWI technique and was carried out in January 2021. The final research sample consisted of 509 respondents. Tourism has always been exposed to various threats: natural disasters, terrorism, etc. In such cases, tourists choose a different, safe direction. However, in 2020, tourism found itself facing a crisis that brought it to a complete stop worldwide. The spread of the COVID-19 virus and safety concerns, as well as global travel restrictions, led to a change in travel during this time. The results show that the respondents resigned especially from going abroad for security reasons, choosing to rather stay in the country and other places that, from their point of view, were safer places to rest.

## 1. Introduction

Tourism is a multidimensional phenomenon considered in many contexts: cultural, spatial, economic, psychological, and social [1]. As Urry [2] put it, being a tourist is one of the determinants of being modern. He claims that going nowhere is like not having a car or a nice house. In modern society, tourism has become a status symbol and is considered a prerequisite for health. Thanks to socio-economic development, and an increase in the level and quality of life, it is becoming a basic need. In wealthy countries, it is considered the fourth measure of the quality of life—after work, housing, and car [3]. Tourism also guarantees psycho-physical regeneration, favouring rest, entertainment, and cognitive self-fulfilment [4]. 

However, tourism is exposed and vulnerable to various environmental, political, and socio-economic threats. Having faced many situations of these types, this area of activity has managed to get used to and even become immune to their influence [5]. 

However, it was only the COVID-19 pandemic situation that prompted the drastic changes in both demand and supply, globally and domestically. In 2020, there were 1 billion fewer international visitors to all destinations around the world than in 2019, due to an unprecedented decline in tourism demand prompted by widespread travel restrictions [6]. 

The complete halt of tourist traffic has affected virtually every single country across the globe. The COVID-19 outbreak led to an unprecedented lockdown that saw more than 3.9 billion people—or half of the world’s population—quarantined and travel-restricted by governments to mitigate the spread of the pandemic [7].

The impact of restrictions on leisure trips is reflected in the data of the UNWTO World Tourism Barometer, which recorded a decrease in foreign trips by 72% in the period from January to October 2020 compared with the same period in 2019 [8].

The COVID-19 pandemic also caused changes in travel behaviour. The changes included the complete abandonment of leisure travel due to the pandemic [9,10], the perception of travel as risky, and low consumer confidence [11,12]. The main source of concern about travel safety is perceived risk, which is a situation characterised by an increased likelihood of harm to health or property [13].

In the second half of 2020 and the first half of 2021, borders were gradually opened for tourists, and air traffic was resumed. However, in many countries, travel restrictions are still in place in order to control the spread of COVID-19. Numerous countries have adopted measures, unprecedented since the end of World War II, in order to stop the spread of the virus and reduce the number of cases and deaths [14]. 

When it comes to COVID-19, EU countries started lifting travel bans as of 1 July 2020, to provide a much-needed boost to economies, especially with regard to the tourism industry that was absolutely demolished by the virus [15]. In various countries, governments have decided to ease restrictions and bring back domestic travel, e.g., for tourist purposes, whilst limiting foreign arrivals. 

The purpose of this article is to identify the impact of the COVID-19 pandemic on Poles’ perception of travel risk and to analyse their travel behaviour during the pandemic.

The following research questions were addressed by this study:

1. For which groups of Polish tourists did the COVID-19 pandemic become a factor limiting tourist activity?
2. How often, when, and how long did the departures take place for?
3. What were the main goals and destinations of Polish tourists?
4. What accommodation facilities did tourists use and how did they meet their nutritional needs?

The structure of this study is as follows. In the first part, we provide a literature review that consists of two parts. In the next section, we present a short description of consumer behaviour in tourism. In another part, we present stressful situations in tourism that took place but did not cause such a shock as the emergence of the COVID-19 pandemic. The next section describes the research methodology. We chose the diagnostic survey method that was carried out with the help of a questionnaire distributed via the Internet. The next section describes our results and discussions, and the last section contains conclusions and recommendations.

At the time of the conceptualisation of the research, the issues related to the research topic had not yet been sufficiently recognised; therefore, the researchers took up this issue.

The research results presented in the article contribute to the discussion undertaken by many researchers. We also included elements that other researchers did not consider, such as tourists’ perceptions of compliance with hygiene standards by different travel service providers, which adds to the knowledge in this area. The research presented in the article concerns the experiences of Poles travelling for leisure in the summer season of 2020, taking into account the context of the COVID-19 pandemic.

## 2. Theoretical Background

### 2.1. Consumer Behaviour in Tourism

The process in which a consumer decides to acquire or use a product or service is defined as the process of consumer behaviour [16]. Consumer behaviour is a set of specific decisions, actions, ideas, and experiences that satisfy consumers’ needs and wants [17]. Consumer behaviour study identifies why people buy a given product and how they make a decision [18]. Kotler and Keller [19] claim that consumer behaviour research is a study of how individuals, groups, and organisations buy and dispose of goods, services, ideas, or experiences to satisfy their needs and wants. Enis [20] defined buyer behaviour as a process, which through inputs and their use leads to the satisfaction of needs and wants. Consumer behaviour is quite complex, since every single customer has a specific way of purchasing, consuming, and disposing of a product. Understanding beliefs and principles concerning customer behaviour allows one to sell goods and services more effectively [21]. The notion of needs and wants is particularly relevant in this process [17]. The behaviour of tourists as consumers is conditioned by many factors [22], which are presented in Table 1. 

Another division of factors influencing customers’ buying behaviour implies distinguishing between two groups, i.e., factors of an internal nature (needs and desires, attitudes and preferences, personality, learning (experience), and risks associated with buying (goods or services)) and external factors (available offers, promotions, advertising, opinions on the Internet, etc.) [27]. In the era of the COVID-19 pandemic, special attention should be paid to the second group, especially with regard to the risk associated with the purchase decision. Risk is most often a subjective measure of assessing the consequences of the occurrence (or not) of a decision-making event under conditions beyond the control of the consumer. These can be categorised as follows:

- Functional (risks related to the functions of the product/service);
- Physical (safety concerns);
- Economic (related to doubts about the price of the product/service);
- Social (related to the acceptance of social groups, e.g., family, friends);
- Psychological (related to the formation of one’s image, prestige, dignity, etc.).

Risk can also be in the form of a waste of time [31].

It is worth noting that there is a division of factors determining the behaviour of tourists, in addition to the previously distinguished consideration of political and so-called other factors, which are often almost impossible to control [32,33]. The policies of the governments of individual countries in the era of the COVID-19 pandemic determined numerous restrictions on the operation of the tourist services sector, but also limited the mobility of its potential customers, causing additional economic difficulties for those operating in the industry. Cultural factors and the natural environment are also of great importance. 

There are five phases in the purchasing decision process:

- Awareness of the need;
- Search for information;
- Evaluation of choice alternatives;
- Purchase decision and purchase;
- Feelings after purchase [34].

The consumer decision-making process includes five stages that a consumer goes through before making a purchase [35]. Not all consumers go through all of the processes; some stages may be skipped or rearranged [36]. However, when consumers are facing a new and complex purchasing situation, they go through all of the processes, for example, buying a tourist product [37]. 

The factors affecting consumer behaviour can also be divided into three categories [38]: (*i*)—consists of personal factors, such as the tourist’s personality, self-image, attitude, motivation, perception, lifestyle, age, family lifestyle, and occupation; (*ii*)—consists of social factors, such as culture, family, social class, and reference groups; (*iii*)—situational factors, such as time, physical and social environment, and state of mind. 

Changes in consumer behaviour can also be addressed in a theoretical context, for example, from the perspective of changing roles and in terms of stress [39]. Thoits [40] defines stress as a set of environmental, social, or internal factors that require an individual to adjust their daily behavioural patterns to new situations and circumstances. As a result of these demands, more or less balanced states that an individual has managed to maintain in his or her life before experiencing stress are typically disrupted, which creates the need for a change in behaviour or mental state [39] and usually causes discomfort resulting from the necessity to adjust to a new situation. Major life changes and transitions are often perceived as “stressors” that create a general need in an individual to adjust [41]. Stressful life events also lead to an initiation, intensification, or change in consumption habits in order to cope with stress [39]. It is completely understandable that in a stressful situation (the COVID-19 pandemic can undoubtedly be considered to be one) the decisions and destination choices of tourists are influenced by their sense of security [42]. 

Participation in tourism is primarily a conscious activity of an individual, focused on values. Tourism is a journey in time and space, but it is also a feature of the human condition, and a motive for cognitive and emotional experiences [43]. Tourist activity is an opportunity to search for and identify a chosen system of values that gives shape and meaning to human life [44]. Agency is one of the basic concepts of the approach to well-being and human development. Efficiency is the ability of a person to pursue goals and act in order to achieve them in accordance with their values. Agency reflects the ability of individuals and groups to effectively shape their own destiny and help each other, and to be active participants in the process of change, not passive and obedient recipients of instructions or assistance. This concept of agency makes people the driving forces of their own development [45].

While the agency theory is essentially an obedience theory, it assumes that people (e.g., tourists) will obey an authority (e.g., a government) if they find it morally right and/or lawful [7,46]. One can also quote here the reactance theory, which states that when personal freedom is limited, eliminated, or threatened, the person then experiences an unpleasant state of arousal (reactivity), which triggers attempts to regain or restore the lost or threatened behaviour. This is due to the fact that individuals have a natural predisposition to preserve and restore personal freedoms [47]. According to this theory, people (in this case, tourists) must freely choose their behaviour (i.e., the need for self-determination) and those people will react to regain this freedom when it is violated, limited, or threatened, e.g., by blockades or traffic control orders [48].

It should be noted that global tourism has been exposed to many different crises in the past [49], which have influenced the behaviour of tourists to varying degrees. Risk-averse consumption behaviours have been observed after some large-scale events: terrorism (September 11) and its impact on the airline industry and tourism worldwide; tsunamis in Thailand, Indonesia, and Japan; earthquakes in Haiti, Japan, and New Zealand; severe flooding and damage in New Orleans and Queensland; pandemics such as SARS and the H1N1 virus; fires in California and Victoria [50]; and the Ebola and Zika viruses [51]. Crises can cause significant changes in tourism; however, none of them have managed to completely crash it so far. Nevertheless, Assaf and Scuderi [52] pointed out that the tourism industry has faced many crises in the past, but the current one caused by the COVID-19 pandemic definitely remains the most harmful one to date. 

Faulkner [53] defines a crisis as an act or omission of an action that interferes with the current functions of an organisation, the acceptable achievement of its goals, or its viability or survival, or which has a detrimental personal impact as perceived by most of its employees or customers. Richter [54] points out that one of the inevitable consequences of globalisation was the increase in international travel and the emergence of infectious diseases. The protection motivation theory (PMT) assumes that when faced with impending events, such as environmental threats or disasters, individuals change their behaviour to protect themselves; however, this depends on three factors: the severity of the risk, the likelihood that the risk can affect them, and the expected effectiveness of the protective response [55].

### 2.2. COVID-19 and Tourism 

A person’s life is made up of various events. Some of them, e.g., going to the dentist or passing a driving license exam, can be described as ordinary, daily, and scheduled. However, there are also extreme events (e.g., a strong earthquake or an outbreak of an infectious disease) that are surprising and can be potentially stressful [56]. An example of the latter was the COVID-19 pandemic, which significantly affected the tourism sector and tourist behaviour. Travel restrictions introduced worldwide, as well as the risk of infection with the SARS-CoV-2 virus, influenced the decisions of both those who had just travelled and those who were in the planning stage.

Such a situation was caused by the global COVID-19 pandemic. The SARS-CoV-2 virus literally isolated societies in their own houses, impeding not only the activities of individuals but entire domestic economies. 

Severe acute respiratory syndrome coronavirus (SARS-CoV-2) is the causal agent of coronavirus disease (COVID-19), a respiratory infection that emerged in Wuhan province of China in late 2019, becoming a global pandemic in 2020 [57]. This unprecedented situation has had several consequences for the everyday life of consumers and has dramatically changed the way that businesses act and consumers behave [58]. 

The COVID-19 pandemic very quickly changed life around the world. In 2020, societies all over the world were blocked, and citizens were requested to respect social distancing and remain at home. To limit the spread of COVID-19, governments across the globe took drastic measures by locking down whole countries or the most affected cities and also by closing their borders, which resulted in an immense hit for the global tourism industry, particularly for the travel and hospitality sectors [59].

Tourism, previously considered to be one of the largest and fastest-growing industries in the world, is currently one of the sectors that has been most adversely and lastingly affected by the pandemic [60,61]. According to UNWTO, international arrivals dropped by 74%. In 2020, the number of tourists worldwide decreased by 1 billion compared to the previous year. This was caused by an unprecedented recession in demand and general travel restrictions [62].

The coronavirus pandemic that began in 2019 has affected people and businesses around the world, causing a global economic crisis, which has also affected the tourism industry. The pandemic has not only affected wages but has also led to various regional changes, including employment opportunities, thereby disrupting the lives of local communities as a whole [63,64]. The scale of impact of COVID-19 may potentially cause long-term changes in spending patterns and industry and consumer behaviour that require special attention in modelling tasks. For instance, the loss of employment resulted in a lower propensity to consume in all households [65].

The state of the tourism industry largely depends on the decisions made by the tourists, which are reflected in their travel behaviour [66]. Consumer behaviour in the tourism market is a very important issue. Understanding it is crucial for the development and conceptualisation of current and new tourism products, as well as the forecasting of the decision-making process in tourism by respective market segments [38] and various tourism businesses, governing bodies, local authorities, and governments. Learning about these processes is particularly relevant in the field of tourism since consumer decisions are often dictated by emotions [18] and are often made spontaneously and impulsively; therefore, it is difficult to explain them based on rational premises.

## 3. Research Method

The study takes the risk perception attitude [67], the health belief model [68,69,70], and the theory of planned behaviour [71,72,73] as the theoretical frameworks. The perception of risk and the belief that one is susceptible to disease can be important factors in self-protective behaviour and reasons for a person to take appropriate preventive measures. Increased risk perception may inhibit or modify actions due to excessive fear caused by risk. The perceived risk of a disease is therefore a driver of changes. These changes can be equated with health behaviours. These are behaviours that may (also in terms of subjective opinion) have an impact on the physical health of a given person. Perceived health risks are one of the most important motives for encouraging pro-health behaviour. Thus, it can be assumed that a person who avoids health risks expects that a specific pro-health behaviour can minimise such risks. This is true of the theory of planned behaviour, which focuses on the influence of subjective norms and individual attitudes on people’s behaviour, which can minimise risk and dangerous situations for their health. An expression of such prevention may be to minimise contact with other people and avoid human concentrations, which may contribute to limiting or completely abandoning tourist trips during a pandemic. Tourist travel during the COVID-19 pandemic is a complex and often quite long-lasting process that inherently carries some level of risk and uncertainty. There are many situations when travelling where the possibility of infection is greater than if you are staying at home. This is why in our research we try to identify the impact of the COVID-19 pandemic on the perception of risk associated with tourist travel and behaviour during these trips.

The diagnostic survey method was implemented in this study. The method of choice was a survey distributed via the Internet. The reason for selecting this technique was the fact that it enabled reaching a large number of people in order to collect the desired data. Due to the purpose of the study, the target group of respondents was to consist of people who partake in tourism activities—hence, purposeful selection was implemented. The study included Polish residents aged 18 years and older. 

We identified a research gap on this subject in the region of Central and Eastern Europe, where Poland is the largest country. So far, many works from China and Asian countries have been published, as well as a few works about Poland [74,75,76]. Tourism-themed groups on Facebook were used in the study. Respondents recruited through Facebook were also asked to offer the survey invitation to fellow tourists via various social media platforms. The research was conducted among people practicing tourism, not among the general public. Hence, we took the decision to use thematic groups focused on tourism. Respondents from all 16 provinces took part in the survey. As a result, our sample is cross-sectional. Thus, the snowball sampling method was used in the recruitment process [77,78,79,80], which significantly increased the reach of the study.

The survey was carried out using webankieta.pl between 4 and 31 January 2021. Determining the size of the study sample is a very important matter in research. According to J.T. Roscoe (as cited in [81]), in the majority of tourism research, an appropriate sample group size should range between 30 and 500 subjects. The final research sample consisted of 509 respondents.

We took into account the following formula for the sample size:

(1) $$n=\frac{P\left(1-P\right)}{\frac{e^{2}}{Z_{\alpha /2}^{2}}+\frac{P\left(1-P\right)}{N}}$$

where *n* is the sample size, *e* is a permissible error, *N* is the population size, and Z_α/2_ is the value resulting from the confidence interval used; for a 95% confidence level, Z_α/2_ = 1.96, and *P* is the estimated proportion in the population (usually, it is set at *P* = 50%). *P* is the estimated expected proportion of the population covered by the study. As the proportion in the population of operators was unknown, the least favourable assumption was made, namely that *P* = 50%, because at that *P* level the product *P*(1 − *P*) reaches the maximum value. It can be assumed that the maximum error of our measurements is 4% (with the significance level α = 0.95).

The data collected from questionnaires were passed on to the statistical analysis stage, conducted with the use of STATISTICA 13.3. software. The survey questionnaire was adapted from earlier studies [76]. In this case, it consisted of 27 questions. The questions included 3 thematic parts. The first concerned the general characteristics of trips, e.g., the purpose and directions of trips, the length and frequency, preferred types of accommodation facilities, and ways of satisfying nutritional needs, as well as the reasons for the lack of tourist trips. The second part of the questionnaire concerned the impact of the COVID-19 pandemic on respondents’ tourist trips. The focus here was on the impact of the pandemic on the implementation of tourist plans, the safety assessment of various types of accommodation facilities, activities undertaken by these facilities to improve safety, and the sources of discomfort during trips in the pandemic. The third part of the questionnaire (7 questions) concerned the characteristics of the respondents.

In the questionnaire, we used single-choice and multiple-choice questions, as well as a four-point Likert scale (1—not important, 4—very important) and a five-point Likert scale (1—strongly disagree, 5—strongly agree).

Cronbach’s alpha formula was applied to assess the reliability and accuracy of data gathered from the surveys. Additionally, non-parametric statistical procedures were also implemented: the Mann–Whitney U test, Wald–Wolfowitz runs test, Kruskal–Wallis H test, chi-square test of independence, and cluster analysis. Significant results were classified as *p* < 0.05 [82,83].

## 4. Results of the Research

The source material consisted of 509 admissible questionnaires completed by respondents. Descriptive statistics indicated that female participants (57.8%) slightly outnumbered male participants (42.2%). The age of respondents varied, with the youngest being 18 and the oldest being 81. The average age was 39.7, whereas the median age was 38. The largest proportion of the sample was between 30 and 39 (31.0%). 

Respondents were well educated—69.0% of them completed university-level education and 28.5% were high school graduates. The high level of education directly affected the positive assessment of respondents’ financial situations. As many as 14.3% of the respondents assessed it as being very good and 59.1% assessed it as being good. In contrast, just over a quarter (25.3%) of the respondents defined their situation as being bearable. It is worth noting that a few individuals described their financial situation as being poor or very poor. 

In addition, in 56.4% of the respondents, the ongoing COVID-19 pandemic did not affect their financial situation. However, its deterioration was claimed by 31.4% of the respondents and its improvement was claimed by 3.7%.

Considering the place of residence, the vast majority (67.4%) was based in urban areas. Over half of them (56.0%) lived in towns with over 100,000 residents, with 16.0% in towns with 50,000 to 100,000 residents, and the rest lived in smaller cities. Rural residents made up 32.6% of the focus group.

Undoubtedly, one of the important factors determining tourist activity is having children. Among those surveyed, 36.5% had children who were below 18.

Table 2 shows the characteristics of those surveyed divided into two groups—the first one consists of those who travelled in 2020, and the second one consists of those who did not. The chi-square test of independence indicated that statistically significant characteristics differentiating between the designated groups included the place of residence (χ^2^ = 17.76, *p* = 0.000) and the age of the participants (χ^2^ = 26.01, *p* = 0.000).

Individuals who did not take any tourist trips in 2020 were much more frequently the residents of rural areas or relatively small towns. Additionally, there was a larger proportion of elderly people in this group, and they were at increased risk of severe illness from SARS-CoV-2. 

No further statistically significant variables were found. 

The description of our research was divided into two sections. The first section consisted of respondents who in 2020 did not partake in any form of tourism. These individuals made up 24.2% of those surveyed and their general characteristics are shown in Table 1. Table 3 represents the descriptive statistics of the reasons for not participating in any form of tourism activities. Cronbach’s alpha coefficient reached 0.732, exceeding the threshold value of 0.7 [71,72], which indicates that all of the scales were internally consistent and appropriate.

Our results indicate that the most significant reason for not participating in any tourism activities was the prevailing COVID-19 pandemic and the fear of contracting SARS-CoV-2 virus during the trip (3.44). 

A lack of time due to work or home duties as well as a lack of sufficient funds to cover the expenses of the trip were among the most frequently mentioned reasons. This appears to be supported by the fact that almost 37% of those surveyed claimed that the pandemic negatively impacted their financial situation. For a significant proportion of those surveyed, a very important reason was the lack of need or desire to travel (2.20), as well as the decision to spend the holiday close to home. 

Given the demographic characteristics of the respondents (gender, education, and age), there was a statistically significant effect of gender on the significance rating of the reason related to official and occupational obligations (Wald–Wolfowitz runs test, *p* = 0.041), whereas the Kruskal–Wallis H test indicated a statistically significant effect of age on the significance rating of the fear of coronavirus infection (*p* = 0.015) and the reason of official and occupational obligations (*p* = 0.007). 

**Figure 1:**
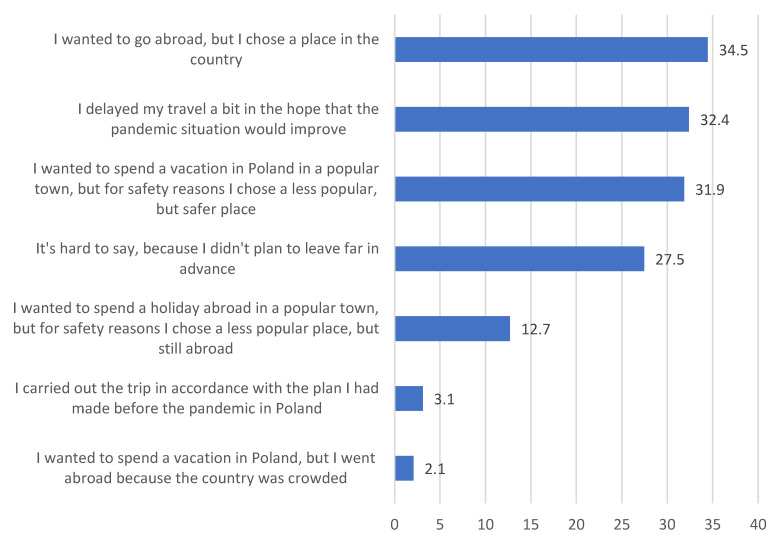
The impact of the COVID-19 pandemic on the change in travel plans of the respondents in 2020 (in %, *n* = 386). The respondents could indicate more than one answer. Source: self-conducted research.

As mentioned before, the main purpose of the study was to determine the level of tourist activities of Poles during the COVID-19 pandemic. Based on the conducted study, it was found that in 2020, 386 out of 509 respondents decided to travel, which represented 75.8% of all of those surveyed. We begin our deliberations about the characteristics of these trips by considering the impact of the COVID-19 pandemic on changes in travel plans in 2020. As shown in Figure 1, a significant number of the respondents adjusted their holiday plans to the COVID-19 situation. These changes mainly included choosing a domestic destination instead of a foreign one (34.5%) or shifting from a trendy tourist resort to a less popular location (31.9%). In addition, a significant number of the respondents decided to postpone their trip, hoping for an improvement in the situation and a substantial drop in the number of cases.

A large proportion of the respondents also indicated that they did not prepare their travel plans very far ahead (the state of epidemic was announced in Poland on 20 March); hence, it was hard to unequivocally determine the impact of the pandemic on their travel plans since they were not yet in place at the time. 

As for travel destinations, domestic trips were clearly predominant among those surveyed. They were selected by 98.4% of the surveyed group. On the other hand, 24.9% of the respondents took a trip abroad. Domestic trips were most often taken twice (36.0%) and once (29.0%), whereas foreign trips were taken once (20.2%) and twice (3.1%). 

**Figure 2:**
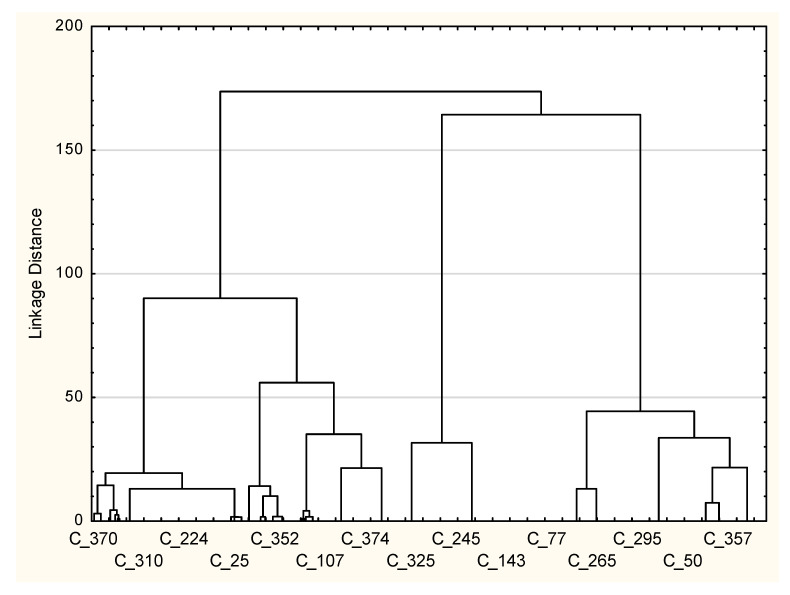
Three-cluster solution: Ward’s method with squared Euclidean distance measures.

Cluster analysis was performed on the scores of the impact of the COVID-19 pandemic on the change in travel plans (Figure 1). A three-cluster solution was selected as the most discriminatory (Figure 2). The results of the multivariate analyses were used to identify the three clusters and to indicate any significant differences between them (*p* < 0.05). 

Cluster 1, the largest cluster of 178 respondents, includes respondents who had as the main factor of the impact of the COVID-19 pandemic on tourist plans attributes regarding “I wanted to go abroad, but I chose a place in the country”. In turn, Cluster 2 includes respondents who delayed their trip a bit in the hope that the pandemic situation would calm down. On the other hand, Cluster 3 comprises respondents who indicated the option “it’s hard to say, because I didn’t plan to leave far in advance”.

As presented in Table 4, the chi-square independence test indicated that only one characteristic of those surveyed contributed to differentiating between the three clusters (*p* < 0.05). It should be noted that the Kruskal–Wallis H test indicated a statistically significant difference between the three clusters based on age (*p* = 0.046) and the assessment of one’s own material situation (*p* = 0.000) too.

The reason why the vast majority of the surveyed group chose domestic destinations may be influenced by the relatively short duration of such trips. The most common trips lasted 3–4 days (44.3%) or 5–7 days (38.3%). It is worth mentioning that weekend trips also grew in popularity (29.3%). 

When discussing the trip duration, the obvious impact of the prevailing pandemic must also be pointed out. More than 40% of respondents stated that the pandemic led to shortening their stay. Moreover, in many cases, the initial travel plans were broken up into several shorter trips (15.5%). This is similar to the fact that, in the case of the impact of the pandemic on the destination choice, some of those surveyed indicated that they did not plan their trips very far ahead (22%). For 15% of the respondents, the trips were as long as initially intended. 

The influence of coronavirus is also quite evident in the month that was chosen by the surveyed group for travelling. Most of the travel took place in the summer months, especially in August; however, although this was the most common choice, as 64.3% of the respondents took their trips in this month, 47.9% decided to travel in July. September was also a popular choice, with 37.6% of those surveyed leaving for holidays. These results are consistent with the answers of 1/3 of the respondents, who indicated that they deliberately postponed their plans in the hope that the epidemiological situation would improve. The surveyed group were slightly less likely to travel before the summer holidays, with 29.0% of them deciding to do so in June and only 13.0% deciding to do so in May. Given the winter break, some of those surveyed also travelled in February (14.0%). In other months, the proportion of the respondents who travelled did not exceed a few percent.

**Figure 3:**
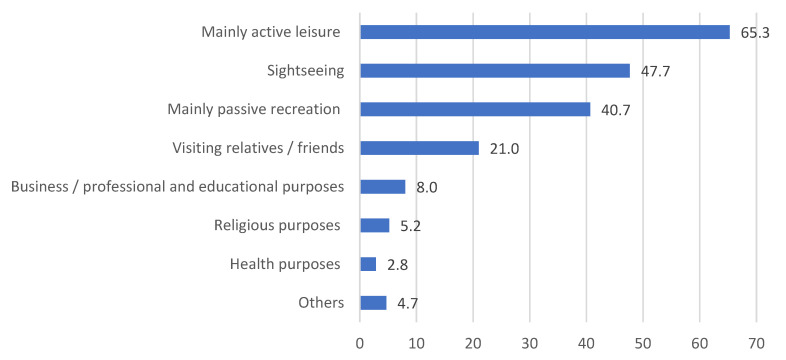
Main purposes of tourist trips among the respondents in 2020. The respondents could indicate more than one answer. Source: self-conducted research.

The most popular purpose of the trips among the surveyed group was active recreation. For many respondents, it was also sightseeing and passive recreation (Figure 3). 

Only about 20% of the surveyed group travelled to visit their relatives or friends. This result was certainly influenced significantly by the prevailing pandemic, which effectively limited and restricted human interaction.

The most common tourist destination among those surveyed was definitely the seaside (over 60%). Frequently chosen destinations were the mountains (40.4%) and lake areas (33.9%). In addition, more popular choices included places considered to be potentially safer from the epidemiological perspective, such as forests (30.3%) and rural areas (22.4%). Such areas are safer mainly due to low tourist traffic. Cities were chosen in 24.4% of respondents.

In the study, we also looked at the travel companions of the respondents. In most cases, it was a partner or a spouse (74.9%), children (39.9%), and friends (37.6%). The percentages of those surveyed accompanied by parents or siblings were, respectively, 10.4% and 8.0%. As much as 7.8% of the surveyed group decided to travel alone. Occasionally, the answers were “other company” (3.6%) and grandchildren (0.8%). 

The important component of the study was to identify the accommodation facilities used by respondents on their excursions in 2020, as well as their opinions on epidemiological safety in these types of facilities. Hotels and resorts were the most common choices due to their high availability. Interestingly enough, these facilities were also considered to be the least safe across all of the categories. Facilities rated as being much safer than hotels were widely popular (from over 20% to almost 40%), including rented apartments, agritourism farms, rented houses/holiday cabins, and private accommodation (rented rooms). Details on this are visible in Table 5. Cronbach’s alpha coefficient reached 0.893. This value indicates the high reliability of the measurement.

The facilities with the best safety ratings, such as a tent or caravan set up somewhere in nature and a holiday cabin/second home, paradoxically were the least used options. The reason behind this is certainly the inconvenience of “squatting” and also the small proportion of individuals who actually own a holiday cabin. It is also worth considering the accommodation type that was less popular and yet was still rated as being relatively safe, namely, an overnight stay with family and friends. While under normal circumstances such a solution would certainly be a more frequently used alternative, this could be quite inconvenient for (probably mainly) hosts and guests, especially in the wake of the pandemic and widespread isolation.

The use of the Mann–Whitney U test showed a significant impact of gender on the assessment of the safety of “Rented apartment” (*p* = 0.014) and “Private accommodation” (*p* = 0.041). In turn, the Wald–Wolfowitz runs test indicated a significant impact of gender on the assessment of safety in the “Camping site” (0.045).

In the study, we also considered different ways of satisfying nutritional needs during excursions. The vast majority of those surveyed chose public eating establishments (66.1%). Another quite common option (52.1%) was self-prepared meals from products purchased in local stores. This choice was available mainly for individuals staying in rented apartments or holiday cabins, i.e., facilities with kitchenettes. Another popular option was half board (e.g., breakfast only) or full board—35.2% and 22.3 %, respectively. As much as 19.2% of those surveyed indicated that they had relied on their own supplies, brought from home.

The pandemic period was certainly not optimal for undertaking tourism activities. The presence of the virus resulted in the introduction of many restrictions and took its toll on many areas of life, including tourism, at times making them less convenient. This was also identified by our respondents, as in their opinion the most bothersome phenomenon was individuals who refused to adhere to mask and social distancing requirements. Quite often, this was to do with too many visitors being in a particular destination, which was also pointed out by our respondents. According to the survey, leisure was disturbed by the introduction of sanitary–epidemiological restrictions in tourist facilities (Table 6).

What is quite interesting is that the fear of the coronavirus infection itself was not popular among the surveyed tourists (an average of 2.77). Some of the surveyed group also pointed out the reduced number of attractions, both within the accommodation facility and the destination itself. Another point raised was the increased prices of tourism services, which potentially were to do with a higher interest in such services due to periodic access restrictions.

**Figure 4:**
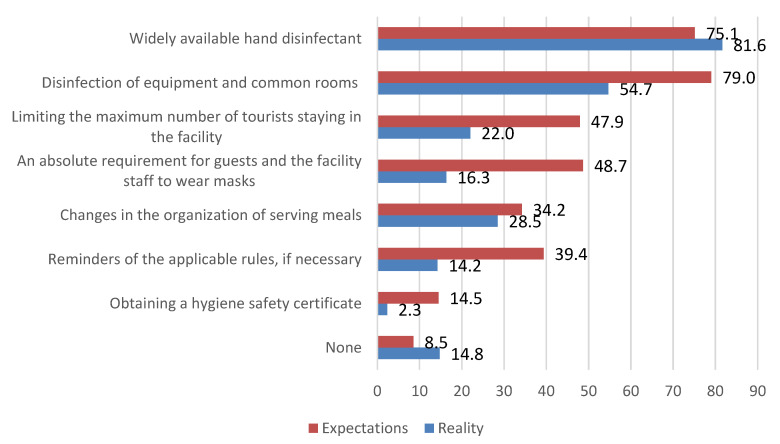
Respondents’ expectations towards measures introduced to improve epidemiological safety by accommodation facilities and actual measures encountered during the stay (in %, *n* = 386). The respondents could indicate more than one answer. Source: self-conducted research.

Given the circumstances, a crucial component of our study was identifying tourists’ expectations towards the accommodation facilities and the actions that said facilities would aim to introduce in order to improve epidemiological safety. We also asked them to describe the measures that they had encountered in the visited accommodation facilities. For the most part, expectations vastly exceeded reality (Figure 4). 

One of the most commonly encountered safety measures in accommodation facilities was hand sanitiser. Unfortunately, the disinfection of tables, doorknobs, and other elements of shared spaces was only observed by about half of those surveyed. It should be underlined that according to the surveyed, mask requirements were mostly ignored by the visitors and personnel in the accommodation facilities, and the staff would hardly ever remind the guests of the prevailing restrictions. 

## 5. Discussion

This study makes an important contribution to the literature on how the COVID-19 pandemic has impacted tourist behaviour. It shows that when concerned about their own safety, tourists adjust their travel and leisure plans. The results of our study support the notion that COVID-19 had an impact on tourist activity in 2020. The surveyed group decided to abandon trips abroad, which were quite difficult anyway due to numerous international restrictions, in favour of domestic leisure travel destinations. Their domestic trips were much more frequent but shorter than usual. Tourists continued to travel, but they reduced the lengths of their stays, and they reprioritised the goods and services that they spent their money on [56].

Perceptions of risk and beliefs about susceptibility to disease can be important factors in self-protective behaviour and reasons for taking appropriate preventive measures, according to the theory of planned behaviour, which focuses on the influence of subjective norms and individual attitudes on people’s behaviour, which can minimise risks and situations that are dangerous to their health. An expression of such prevention can be minimising contact with other people and avoiding concentrations of people, which can contribute to reducing or completely abandoning tourist trips during a pandemic [71,72,73].

The COVID-19 pandemic is the greatest challenge that the modern travel industry has ever faced [84], and the long-term effects of the pandemic, not only for tourism, are yet to come [85]. Ensuring safety is an important part of holiday planning, mainly due to a number of unfamiliar situations that are an integral part of visiting new places and meeting new people [10]. Epidemic crises may provoke important shifts in demand for certain destinations, as travellers may consciously decide not to be exposed to such crises [86]. The COVID-19 pandemic caused many consumers to deviate from their usual shopping habits and even learn some new ones [86]. 

Due to the economic impact of limited/cancelled travel, border closures, and lockdowns, the industry wants to understand potential travellers in order to develop strategies that encourage people to travel again [87].

Another study found [88,89] that the risk of SARS-CoV-2 virus infection negatively affected Chinese travel decisions, particularly regarding the number of trips and the change in destinations. A study by Orindaru et al. [90] found that the COVID-19 pandemic and the decline in outbound travel had a positive impact on domestic tourism. The abandonment of foreign travel is due to a fear of possible complications with returning to the country, the risk of flight suspensions, quarantine obligations, etc. [91].

As our study shows, the COVID-19 situation demonstrates a change in typical vacation behaviour, and has caused a significant drop in tourist numbers [10]. Travel anxiety caused by the COVID-19 pandemic leads to abandoning holiday plans altogether, foregoing overseas travel [92], and even reducing the duration of planned holidays [93]. Risk perception influences destination selection; however, the level of perceived risk only leads to destination swaps and not to a permanent end to all international travel plans [94].

However, people were forced to stay in the country and chose to satisfy their travel needs through domestic tourism. Therefore, domestic markets have now become the biggest hope for the tourism industry [95]. Relatively minimal domestic travel restrictions combined with difficulties or complete bans of global tourism caused domestic tourism to become the only viable option for those wishing to partake in tourism activities [96].

The pandemic situation had an impact on the everyday life of consumers and changed the way that companies and consumers operated [58]. The outbreak of the global pandemic has already caused a number of changes in shopping behaviour, and we can only wonder how long they will last and whether they will stay with us after the pandemic is over [97].

COVID-19 will change tourists’ habits and preferences toward the practice of more outdoor activities with the purpose of social distancing and more sustainable forms of tourism [55]. According to WTTC, once the pandemic is over, it will take over a year for the travel and tourism industry to regain its previous levels of participation, if it even does [50]. The COVID-19 pandemic will most likely cause a long-term paradigm shift in the world, even after it is over [52].

COVID-19 crippled global tourism and provided the opportunity to stop tourism activities and think about the consequences. Changes in the tourism industry brought about by the prevailing pandemic have undoubtedly highlighted the roles and significance of safety both for tourists and service providers, which in turn will require procedural and distributive changes to inform any restorative action for a responsible and just recovery [98]. Similar global crises may become more frequent and must be tackled across different areas of economic activity [99].

## 6. Conclusions

The present study adds to the still sparse literature by conducting an empirical study on the impact of COVID-19 on consumer behaviour in the tourism industry. Our research and that of different authors have shown that in the face of this worldwide threat, tourism and tourists are powerless and are forced to revise their plans. It is important to study the factors that affect tourists’ safety in holiday destinations.

Our research (through the research questions posed) has identified the following:

- The group of Polish tourists who, in the conditions of the COVID-19 pandemic, most often abandoned their planned tourist trips were elderly people, usually from rural areas, who feared both infection and organisational difficulties or increased travel costs (resulting from stricter sanitary requirements)—these tourists compensated for the lack of foreign trips with domestic stays;
- Fears caused by the pandemic, and initially also contradictory information on its mechanisms, caused Polish tourists’ decisions to be influenced by factors such as the length of stay (a tendency to shorten trips), time of year (the possibility of staying outside as long as possible), and location (proven or reputable, usually close to the place of residence);
- During the COVID-19 pandemic, Polish tourists chose to travel in the company of members of their own families, using facilities allowing the recommended social distance, often deciding, for safety reasons, to prepare meals themselves in the selected accommodation (which was also an important factor in its selection).

It can therefore be concluded that the results of our research are consistent with the theory of risk perception, the model of health beliefs, and the theory of planned behaviour.

It also turned out that the majority of respondents planning a tourist trip decided to spend it in their country. The decision to change the destination was influenced by demographic and social factors. During the COVID-19 pandemic, respondents decided to travel in the company of their own family, using facilities that allowed them to maintain the recommended epidemiological safety. It is worth emphasising that the surveyed respondents considered renting an apartment and private accommodation as being the safest in terms of contracting the Sars-co2 virus.

Our research related to tourist behaviour during the pandemic period is part of a trend that has been well observed for years, which can be summarised as ‘luxury in nature’. However, this concept has been extended to include the multi-faceted issue of safety, which no longer only includes access to basic technical infrastructure, especially sanitation, but also comprehensive facilities for preparing and serving meals. This is a challenge for the tourism sector as a whole, as well as for the authorities. With understanding and diligent care for said factors from the local authorities, service providers, and travel agencies, tourists might be more inclined to choose these and no other, less safe, travel destinations. Additionally, future tourism and travelling habits may change precisely because of safety concerns among tourists. The main focus should be placed on tourism demand research and analysing different scenarios with different factors that may affect travel in the future [100].

This research has some limitations. Among them, the sample size can be indicated. Increasing it could contribute to reducing the maximum error of our measurements. The limitation also stems from the fact that we conducted the research via the Internet, including Facebook travel groups. Not everyone uses Facebook, and some may not even have access to the Internet. Among such people, we can mention, for example, the elderly. Seniors are numerous and thus constitute an important segment of the tourist market; therefore, it seems interesting and advisable to conduct research only among the elderly. The next problem may be closed questions (some answer options are missing) and the respondents’ bias. We would also like to conduct similar research in an international con-text, which is why we invite scientists from other countries taking up similar research problems to cooperate with us.

The findings of this study can help managers of tourism companies but also destination management organisations to better understand tourist behaviour in the face of various biological threats, also with potential future epidemics in mind.

Policymakers and tourism practitioners need to develop a new preparedness and response mechanism for future crises, but also one to combat the effects of the ongoing pandemic. In addition, it is important to conduct research on travellers’ behaviour after the end of the pandemic, and assess how their plans were affected by the COVID-19 vaccination process.

## Figures and Tables

**Table 1:** Factors affecting consumer behaviour in the tourism market.

Economic		Non-Economic			
outside	inside	psychological	personal– demographic	social–cultural:	others
- product - price - place sale - advertisement	- income - savings and credits - free time	- personality - processes, cognitive - processes, activating	- age - sex - education - healthy - lifestyle	- family - reference groups and leaders opinions - classes and layers of social - culture and subculture	- political - legal - natural - ecological

Source: own study based on [23,24,25,26,27,28,29,30].

**Table 2:** Characteristics of the examined group with regard to individuals participating and not participating in tourism activities in 2020 (*n* = 509).

Characteristic	Individuals Participating in Tourism Activities *n* = 386	Individuals not Participating in Tourism Activities *n* = 123
	**(in %)**	
**Gender**		
female	58.3	56.1
male	41.7	43.9
**Education**		
basic vocational or lower education	2.1	4.1
upper secondary education	26.9	33.3
higher education	71.0	62.6
**Assessment of one’s own material situation**		
very good	14.3	14.6
good	61.4	52.0
bearable	23.8	30.1
poor and very poor	0.5	3.3
**The impact of the pandemic on the respondents’ financial situations**		
remained unchanged	58.0	51.2
deteriorated	29.8	36.6
improved	3.9	3.3
hard to say	8.3	8.9
**Place of residence**		
rural area	28.2	46.3
city with up to 50,000 inhab.	18.7	19.5
city with 50,000–100,000 inhab.	12.7	4.9
city with over 100,000 inhab.	40.4	29.3
**Children (below 18) in a household**		
household without children	61.7	68.3
household with children	38.3	31.7
**Age**		
up to 29	27.2	10.5
30–39	31.9	28.5
40–49	26.2	29.3
50 and more	14.7	31.7
	(number of years)	
the average age	38.0	44.9
the median age	37	44

Source: self-conducted research.

**Table 3:** Reasons preventing or discouraging the respondents from taking trips in 2020 (*n* = 123).

Reason	Not Important	Slightly Important	Moderately Important	Very Important	Average (a Scale of 1–4)	Standard Deviation
	(in %)					
The pandemic, fear of coronavirus infection	4.1	8.9	26.0	61.0	3.44	0.82
Lack of time	24.4	30.9	30.1	14.6	2.35	1.01
Official and occupational obligations	30.9	30.1	26.0	13.0	2.21	1.03
No need/desire to leave	34.1	26.0	26.0	13.8	2.20	1.06
I prefer to relax where I live	34.1	25.2	28.5	12.2	2.19	1.04
Insufficient funds for such trip	35.0	29.3	18.7	17.1	2.18	1.09
Household duties	30.1	38.2	18.7	13.0	2.15	1.00
Inability to book an accommodation facility on the preferred date	40.7	31.7	15.4	12.2	1.99	1.03
Organisation issues	48.8	27.6	13.0	10.6	1.85	1.01
No suitable offer	46.3	32.5	16.3	4.9	1.80	0.89
Impossible due to health condition	56.1	26.0	13.8	4.1	1.66	0.87
No company	54.5	30.9	8.9	5.7	1.66	0.87

Source: self-conducted research.

**Table 4:** Profile of the clusters.

Variables	Cluster 1 *n* = 178	Cluster 2 *n* = 96	Cluster 3 *n* = 112
Gender	Female	Female	Female
(χ2 = 1.74; *p* = 0.419)	(61.8%)	(52.8%)	(54.5%)
Level of education	Higher education	Higher education	Higher education
(χ2 = 3.86; *p* = 0.145)	(66.8%)	(78.1%)	(71.4%)
Assessment of one’s own material situation	Good	Good	Good
(χ2 = 14.77; *p* = 0.005) *	(56.7%)	(71.9%)	(59.8%)
Age	30–39	40–49	30–39
(χ2 = 8.67; *p* = 0.193)	(30.9%)	(33.3%)	(32.1%)

* Statistically significant differences: *p* ≤ 0.05.

**Table 5:** Types of accommodation facilities used by the surveyed group and epidemiological safety rating of said facilities (in %, *n* = 386).

	Percentage of Facility Visitors Among the Respondents	Epidemiological Safety Rating of the Facility (on a Scale of 1 to 5)	Standard Deviation
Hotel or holiday resort	46.1	2.79	1.03
Rented apartment	36.5	4.15	0.72
Agritourism farm	27.5	3.70	0.79
Rented house/holiday cabin	22.3	4.27	0.82
Private accommodation—rented room	20.5	3.48	0.94
Overnight stay with family/friends	16.8	3.87	0.99
Guesthouse	11.4	3.14	0.89
Camping site (a tent, a caravan)	7.5	4.06	0.91
“Squatting” in a tent, caravan	4.4	4.61	0.77
Owning a holiday cabin/second home	3.9	4.89	0.36

The respondents could indicate more than one answer. Source: self-conducted research.

**Table 6:** Pandemic-related sources of discomfort among the respondents during their excursions in 2020 (in %, *n* = 386).

	Not Important	Slightly Important	Moderately Important	Very Important	Average on a Scale of 1 to 4	Standard Deviation
	(in %)					
Individuals who refused to adhere to mask and social distancing requirements	10.9	14.2	27.5	42.7	3.07	1.03
Discomfort caused by the introduction of sanitary–epidemiological restrictions in tourist facilities	10.1	25.1	32.4	29.0	2.83	0.98
Overtourism	17.4	19.7	18.9	35.0	2.79	1.15
Fear of coronavirus infection	12.2	25.6	30.8	27.5	2.77	1.00
Reduced number of tourist attractions in the destination	16.3	25.9	24.4	27.2	2.67	1.07
Increased prices of tourism services	12.7	28	27.5	20.5	2.63	0.99
Limited infrastructure and recreational equipment at the accommodation facility	22.5	27.5	19.7	19.7	2.41	1.09
Reduced number of attractions and leisure activities offered by the accommodation facility	23.8	26.4	21.2	18.1	2.38	1.08
Very few tourists—I like meeting new people	47.4	22.5	10.9	4.7	1.68	0.90

“I have no opinion” answers were not included in the table. Source: self-conducted research.

## Data Availability

Data are available from authors upon reasonable request.
